# Comparative Pathogenicity and Transmissibility of Pandemic H1N1, Avian H5N1, and Human H7N9 Influenza Viruses in Tree Shrews

**DOI:** 10.3389/fmicb.2019.02955

**Published:** 2019-12-20

**Authors:** Shuai Xu, Xuyong Li, Jiayun Yang, Zhengxiang Wang, Yane Jia, Lu Han, Liang Wang, Qiyun Zhu

**Affiliations:** State Key Laboratory of Veterinary Etiological Biology, Lanzhou Veterinary Research Institute, Chinese Academy of Agricultural Sciences, Lanzhou, China

**Keywords:** H1N1, H5N1, H7N9, tree shrew, infectivity, transmissibility

## Abstract

Influenza A viruses (IAVs) continuously challenge the poultry industry and human health. Studies of IAVs are still hampered by the availability of suitable animal models. Chinese tree shrews (*Tupaia belangeri chinensis*) are closely related to primates physiologically and genetically, which make them a potential animal model for human diseases. In this study, we comprehensively evaluated infectivity and transmissibility in Chinese tree shrews by using pandemic H1N1 (A/Sichuan/1/2009, pdmH1N1), avian-origin H5N1 (A/Chicken/Gansu/2/2012, H5N1) and early human-origin H7N9 (A/Suzhou/SZ19/2014, H7N9) IAVs. We found that these viruses replicated efficiently in primary tree shrew cells and tree shrews without prior adaption. Pathological lesions in the lungs of the infected tree shrews were severe on day 3 post-inoculation, although clinic symptoms were self-limiting. The pdmH1N1 and H7N9 viruses, but not the H5N1 virus, transmitted among tree shrews by direct contact. Interestingly, we also observed that unadapted H7N9 virus could transmit from tree shrews to naïve guinea pigs. Virus-inoculated tree shrews generated a strong humoral immune response and were protected from challenge with homologous virus. Taken together, our findings suggest the Chinese tree shrew would be a useful mammalian model to study the pathogenesis and transmission of IAVs.

## Introduction

Influenza A viruses (IAVs) are segmented, single-stranded, negative-sense RNA viruses, whose genome comprises eight gene segments, including basic polymerase 2 (PB2), basic polymerase 1 (PB1), acidic polymerase (PA), hemagglutinin (HA), nucleoprotein (NP), neuraminidase (NA), matrix (M), and nonstructural protein (NS). IAVs are divided into 18 HA and 11 NA subtypes on the basis of the antigenicity of their HA and NA surface glycoproteins.

IAVs continuously challenge the poultry industry and human health due to antigenic shift and drift. In the twentieth century, H1N1, H2N2, and H3N2 viruses caused four influenza pandemics in humans, resulting in widespread disease and severe loss of life (Medina and Garcia-Sastre, 2011; Lipsitch, 2013). In 2009, a novel swine-origin influenza A (H1N1) pandemic caused huge economic losses and casualties (Novel Swine-Origin Influenza A (H1N1) Virus Investigation Team et al., 2009). In addition, since 1997, increasing numbers of humans have been infected with highly pathogenic avian influenza H5N1 viruses, with a mortality rate of about 60% among confirmed cases (WHO, 2017). Most recently, avian influenza A (H7N9) viruses have emerged and infected human. To date, the H7N9 avian influenza virus has caused multiple outbreaks of severe disease in humans (Shi et al., 2018; WHO, 2018).

Animal models are essential for studies on the infection, immunity, and transmission of IAVs. Animal models such as mouse, cotton rat, pig, guinea pig, ferret, and nonhuman primate have been extensively developed, but many gaps remain in our understanding (Margine and Krammer, 2014). Each laboratory animal has its advantages and drawbacks. Generally, mouse and guinea pig models are used for pathogenicity and transmission studies. However, these animals do not exhibit some of the clinical symptoms experienced by humans, such as nasal exudates, fever, sneezing, and coughing, and their genetic backgrounds are far from those of humans. Ferrets and nonhuman primates (e.g., macaques) are excellent mammalian animal models for influenza virus pathogenicity and host immunity. Also, ferrets have been used as an important model for influenza virus transmission (Sun et al., 2016, 2019). Moreover, the clinical symptoms of influenza virus-infected ferrets are similar to those of humans (Maines et al., 2006; Shinya et al., 2012). However, availability, cost, livestock requirements, and ethical constraints limit the widespread use of these animals. Therefore, efforts remain focused on the development of new animal models in the influenza field.

The Chinese tree shrew (*Tupaia belangeri chinensis*) is a squirrel-like mammal widely distributing in south and southwest China. It has a small body size (100–150 g), a low maintenance cost, a short reproductive cycle (~6 weeks) and life span (6–8 years), and a much closer affinity to primates than that of ferrets and rodents (Xu et al., 2013). A comparative analysis of the tree shrew and human genome identified 28 genes in the tree shrew genome that were previously considered to be primate-specific (Fan et al., 2013). The high level of similarity in gene sequences between the tree shrew and humans has laid the foundation for the genetic basis to evaluate the tree shrew as a feasible animal model to study related diseases. In contrast, the unique genetic characteristics of tree shrews provide an opportunity for us to understand specific pathways mediated by the unique genes. For instance, loss of the important antiviral gene DDX58/RIG-I (retinoic acid inducible gene I) in the Chinese tree shrew has made the tree shrew a suitable animal model for studying viral infections (Fan et al., 2013; Xu et al., 2016; Yao, 2017).

Since 1970s, tree shrews have been used as animal models for various viruses. Herpes simplex virus (HSV) was the first virus known to infect tree shrews, and tree shrews proved to be a viable model to study HSV latency (Darai et al., 1978; Li et al., 2015, 2016). Moreover, the tree shrew was the only non-primate animal found to be susceptible to hepatitis B virus (HBV) infection, and therefore was used to study HBV infection for many years (Guo et al., 2018). In 2012, the cellular functional receptor of HBV, sodium taurocholate cotransporting polypeptide (NTCP), was identified in tree shrew model (Yan et al., 2012). Tree shrews have been demonstrated to possess all of the essential factors required for hepatitis C virus (HCV) infection and can be used as a potential platform for studying HCV infection (Tong et al., 2011; Feng et al., 2017). Most recently, the tree shrew was used as an animal model to study Zika virus (ZIKV) infection, exhibiting robust viral secretions in sera and saliva as well as cutaneous inflammation and dermatological manifestations, which were similar to those in ZIKV-infected patients (Zhang et al., 2019).

In 2013, Yang et al. analyzed the distribution of ɑ2,3 and ɑ2,6 sialic acid receptors in the respiratory tract of tree shrew (Yang et al., 2013), and investigated the pathological change, seroconversion, and cytokine response of tree shrews infected with H1N1, H9N2 IAVs, and influenza B virus (Yang et al., 2013; Li et al., 2018; Yuan et al., 2019). Most recently, Sanada et al. assessed the pathogenicity of H5N1 and H7N9 IAVs in tree shrews and found that H5N1 influenza virus infection caused severe diffuse pneumonia with fever and weight loss; in contrast, H7N9 influenza virus infection caused focal pneumonia in this tree shrew model (Sanada et al., 2019). These studies suggested that tree shrews could be a useful alternative mammalian model to study the pathogenesis of influenza virus. However, transmissibility and immune responses in the tree shrew model for different IAVs have not been comprehensively investigated.

To better prepare for IAV pandemics and develop more effective prevention and control strategies, a comprehensive understanding of the biological characteristics of the various influenza virus infections is necessary through the use of a suitable animal model. In the present study, we investigated the susceptibility and transmissibility of H1N1, H5N1, and H7N9 IAVs in tree shrews, and assessed the humoral immune response in this model.

## Materials and Methods

### Biosafety and Ethical Statements

All experiments with live influenza viruses were conducted within enhanced animal biosafety level 3+ (ABSL3+) facilities. This study was carried out in strict accordance with the recommendations in the Guide for the Care and Use of Laboratory Animals of the Ministry of Science and Technology of the People’s Republic of China. The details of the facility and the biosafety and biosecurity measures used have been previously reported (Zhang et al., 2013b). The protocols for animal studies were approved by Animal Ethics Committee of Lanzhou Veterinary Research Institute, Chinese Academy of Agricultural Sciences.

### Animals

One to four-month-old female Chinese tree shrews weighing 90–110 g was obtained from the experimental animal core facility of the Kunming Institute of Zoology, Chinese Academy of Sciences (Kunming, China). Six-week-old female Balb/c mice and five- to six-week-old female Hartley strain guinea pigs (250–300 g) were purchased from Vital River Co. Ltd., Beijing, China. All animals were housed in ventilated cages, and provided food and water *ad libitum*.

### Cells and Viruses

Madin-Darby canine kidney (MDCK, American Type Culture Collection, USA) cells were grown in DMEM (Gibco-BRL; 11965-092) supplemented with 10% (vol/vol) FBS (Gibco-BRL; 10099-141) and 1× penicillin/streptomycin (Gibco-BRL; 10378016) at 37°C in 5% CO_2_. Primary renal cells (TSPRCs) and lung cells (TSPLCs) were established from Chinese tree shrews (aged 1–4 months) as follows: kidneys or lungs were taken from the sacrificed tree shrew and minced into small pieces (about 1 mm^3^) in pre-cold PBS, and the pieces were transferred into a 50 ml sterile plastic tube containing 5 mg/ml collagenase type IV (Invitrogen, USA) solution for 30 min in a 37°C water bath. After digestion, the solution was filtered through a 200-mesh sieve to remove tissue pieces. The TSPRCs or TSPLCs were suspended and washed three times with pre-cold PBS. Finally, the cells were resuspended and cultured at a density of 2 × 10^6^ cells/ml in high-glucose DMEM supplemented with 10% FBS and 1× penicillin/streptomycin at 37°C in 5% CO_2_ until confluent. The cells were passaged three times and then used for the indicated experiments.

H1N1 virus A/Sichuan/1/2009 (pdmH1N1) was isolated from the first human case of the 2009 influenza pandemic in China. H5N1 highly pathogenic avian influenza virus A/Chicken/Gansu/2/2012 (H5N1) was isolated in Gansu Province in China in 2012 (Yang et al., 2019). H7N9 virus A/Suzhou/SZ19/2014 (H7N9) was isolated from Jiangsu Province in China in 2014. Virus stocks were propagated in specific-pathogen-free (SPF) chicken eggs and stored at −70°C until use.

### Experimental Infection of Animals

Each animal was inoculated with viruses at 10^6^ EID_50_ in a volume of 200 μl for tree shrews, 50 μl for mice or 300 μl for guinea pigs after they were lightly anesthetized with 0.5% pentobarbital sodium or carbon dioxide. The control animals were inoculated with an equal volume of PBS. Clinical signs of infection and body weight were recorded daily. The animals were sacrificed on the indicated days post-inoculations for virologic and pathological assays.

### Viral Growth Kinetics

TSPRCs and TSPLCs were infected with viruses at a multiplicity of infection (MOI) of 0.01. One hour after infection, the mediums were replaced with fresh OPTI-MEM (containing 0.05 μg/ml TPCK-trypsin) and the cells were maintained at 37°C. Virus-containing culture supernatants were collected at the indicated timepoints, and titrated in eggs. Growth data are presented as the average of three independent experiments.

### Receptor Binding Specificity Assay

We tested the receptor binding specificity of the HA protein for α2,3- or α2,6-linked sialic acid using a solid-phase binding assay with two different glycopolymers: α-2,6 glycans (6′SLN, Neu5Aca2-6Galb1-4GlcNAcb-PAA-biotin) and α-2,3 glycans (3′SLN, Neu5Aca2-3Galb1-4GlcNAcb-PAA-biotin), as previously described (Imai et al., 2012). Briefly, a streptavidin-coated high-binding capacity 96-well plate (Pierce) was incubated with PBS containing different concentrations (starting from 2.4 μM) of biotinylated glycans at 4°C overnight. After the glycan solution was removed, the plates were washed four times with ice-cold PBS and then incubated at 4°C overnight with PBS containing 128 HA units of purified influenza virus. After washing, the plates were incubated for 4 h at 4°C with mouse antibody against influenza NP. The plates were then washed four times and incubated with horseradish peroxidase (HRP)-conjugated goat-anti-mouse antibody (Sigma-Aldrich) for 2 h at 4°C. After four washes, the plates were finally incubated with tetramethylbenzidine substrate (Thermo Scientific), and the reaction was stopped with 50 μl of 2 M H2SO4. Absorbance was determined at 450 nm.

### RNA Isolation and Quantitative PCR

Total RNA from tree shrew primary cells was extracted with TRIzol (Invitrogen), following the manufacturer’s instructions, and was subsequently transcripted into cDNA using M-MLV Reverse Transcriptase, according to the manufacturer’s protocol (Promega). Real-time PCR was carried out using the ABI 7500 detection System (Applied Biosystems, CA). The mRNA level of each cytokine or chemokine was shown as fold of induction (2^−ΔΔCT^) in the graph. The sequences of the gene-specific primers used for qPCR were shown in Table 1.

**Table 1:** Primers for qRT-PCR used in this study.

Primer name	Sequence (5′-3′)
GAPDH-F	TCATTGACCTGAACTACAT
GAPDH-R	GAAGATGGTGATGGACTT
IFNβ-F	CTGAGGAAATTCAACGACCAC
IFNβ-R	ATAATAGCTCTTCAGGTGCAT
IFIT2-F	GGCCAATGGAAATCTCTACCAG
IFIT2-R	AGATAGCCTTTTCTTCGCACT
OASL-F	CTCGAGCTACTAACCATCTACGC
OASL-R	TCTGCCTCCTTCTGCTACGTT

### Virus Titration

Virus titers of virus stocks and homogenized tissue samples were determined by end-point titration in eggs and/or MDCK cells. For end-point viral titration in eggs, 10-fold serial dilutions of each sample were inoculated into eggs. Sixty hours after inoculation, fluid from the allantoic cavity was collected and tested for the ability to agglutinate chicken erythrocytes as an indicator of viral replication. Infectious virus titers are reported as log10 EID_50_/ml, and were calculated from three replicates by the method of Reed-Muench.

### Pathological Analysis

Lung tissue was collected and fixed in 10% neutral buffered formalin for histopathological examination, which was performed as described previously (Masic et al., 2009). Tissue sections of lungs were stained with hematoxylin and eosin (H&E) and examined microscopically for alveolar edema, interstitial edema, hemorrhage, and inflammatory infiltration. The lesion severity of each of four pathological lesions was scored according to the distribution or extent of lesions within the sections examined according to the following scale: 0, no visible changes; 1, mild focal or multifocal change; 2, moderate multifocal change; 3, moderate diffuse change; or 4, severe diffuse change. Two independent pathologists scored all slides from blinded experimental groups.

### Protein Content and Differential Blood Count of Bronchoalveolar Lavage Fluid

The bronchoalveolar lavage fluid (BALF) was obtained from tree shrews on day 3 post-inoculation. BALF was obtained from individual animals *via* tracheal cannulation and lavage with 2 ml of PBS. Protein content as a surrogate metric of lung barrier function was quantified by use of a BCA assay. For BALF cell analysis, BALF was centrifuged at 500 ×*g* for 10 min to spin down the cells and the collected cells were resuspended in 2 ml of PBS. The cell suspension was washed three times in PBS buffer. Differential cell counts were obtained from smears stained with May-Grünwald-Giemsa. At least 200 cells were counted for each animal.

### Antibody Response Assessment

Tree shrews were prime-inoculated intranasally (i.n.) with 10^6^ EID_50_ of test viruses. Sera were collected from all animals 1 day before and on day 14 post-inoculation (p.i.). Twenty-one days post prime-inoculation, the tree shrews were challenged i.n. with 10^6^ EID_50_ of the same virus. Nasal washes were collected from all of the animals at 2-day intervals, beginning on day 2 post-challenge and titrated in eggs. Sera were collected from all tree shrews on day 14 post-challenge for hemagglutinin inhibition (HI) and virus neutralization (VN) tests.

### Intra- or Inter-Species Transmission Study

For the intra-species transmission study, groups of three guinea pigs or tree shrews were inoculated i.n. with 10^6^ EID_50_ of test virus and housed in a ventilated cage. After 24 h, three guinea pigs or tree shrews were cohoused in the same cage as the inoculated animals. For the interspecies transmission study, three animals (tree shrews or guinea pigs) were inoculated i.n. with 10^6^ EID_50_ of test virus and three animals of the other species (guinea pigs or tree shrews) were cohoused in the same cage at 24 h post-inoculation. Body weights of the inoculated and exposed animals were recorded at 2-day intervals, starting on day 0 p.i. Nasal washes were collected from all of the animals at 2-day intervals, starting on day 2 p.i. [1 day post-exposure (p.e.)], which was performed as descripted previously (Lowen et al., 2006). The nasal wash samples were first kept in −80°C and titrated in eggs. Sera were collected from each animal on day 2 before inoculation and day 21 p.i. for HI and VN tests.

### Serological Assays

After serum samples were pretreated with receptor-destroying enzyme to eliminate inhibitors of hemagglutination, serum antibody titers were determined by using the HI test with 0.5% chicken red blood cells (prepared in our laboratory from SPF chickens) and VN in MDCK cells, which were performed as described previously (Maines et al., 2006; Laursen et al., 2018). The cutoff value used for the HI and VN antibody assays was 10.

### Statistical Analysis

The statistical significance of comparisons between two groups was determined with the Student’s *t*-test. *p* less than 0.05 were considered statistically significant. Comparisons of more than two groups were made with ANOVA with Bonferroni corrections. Survival analysis was performed with GraphPad Prism 6.

## Results

### Pandemic H1N1, Avian H5N1, and Human H7N9 Influenza Viruses Efficiently Replicate in Primary Tree Shrew Cells

Yang and his colleagues demonstrated that H1N1 and H9N2 influenza viruses replicate in the upper respiratory tract of tree shrews, and exhibited moderate respiratory symptoms and pathological signs (Yang et al., 2013; Li et al., 2018). In the present study, to characterize the susceptibility of tree shrews to different IAVs, pandemic 2009 H1N1 virus A/Sichuan/1/2009 (pdmH1N1), avian-origin H5N1 virus A/Chicken/Gansu/2/2012 (H5N1), and human-origin H7N9 virus A/Suzhou/SZ19/2014 (H7N9) were selected as representative viruses. We found that the growth and infectivity of all three viruses were comparable in 9-day-old specific-pathogen-free (SPF) chicken eggs, but diverse in MDCK cells (Table 2). Our recent study showed that A/Chicken/Gansu/2/2012 (H5N1) was lethal to chickens and intravenous pathogenicity index was 2.97, indicating that the H5N1 virus was highly pathogenic for chickens (Yang et al., 2019). Molecular characterization indicated that the H5N1 virus possesses a polybasic cleavage site motif (PQRERRRKR/GLF), whereas pdmH1N1 and H7N9 viruses lack this feature (PSIQSR/GLF or PEIPKGR/GLF), suggesting pdmH1N1 and H7N9 viruses may be low pathogenic for chickens (Table 2). Additionally, we tested the receptor-binding properties of three viruses and found that pdmH1N1 virus only bound to α2, 6-siaylglycopolymer (human-type receptor), H5N1 virus only bound to α2, 3-siaylglycopolymer (avian-type receptor), and H7N9 virus bound to both receptors, which had greater affinity with α2, 6-siaylglycopolymer than that with the α2, 3-siaylglycopolymer (Supplementary Figure S1).

**Table 2:** Growth and pathogenicity characteristics of three viruses.

Viruses	Virus growth^a^		HA cleavage site motif	Receptor-binding preference	
	log_10_EID_50_/ml	log_10_TCID_50_/ml		SA α-2, 3	SA α-2, 6
pdmH1N1	8.35 ± 0.13	5.58 ± 0.14	PSIQSR/GLF	No	Yes
H5N1	8.69 ± 0.17	6.64 ± 0.13	PQRERRRKR/GLF	Yes	No
H7N9	8.24 ± 0.08	4.69 ± 0.17	PEIPKGR/GLF	Yes	Yes

a *All data are the mean ± SD from three independent experiments*.

**Figure 1:**
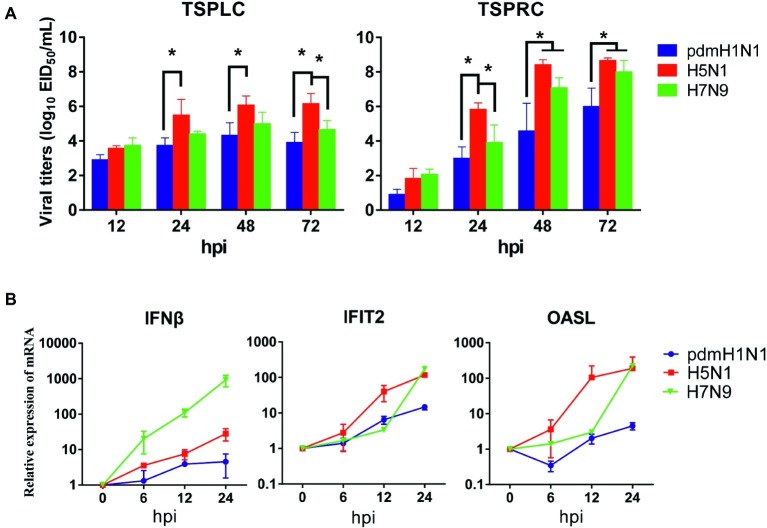
Growth kinetics of influenza viruses and cytokine response in primary tree shrew cells. **(A)** Virus titers in tree shrew primary cells infected with pdmH1N1, H5N1, and H7N9 influenza viruses. Virus growth characteristics were determined by use of multi-cycle growth curves in tree shrew primary lung (left) or renal (right) cells infected with viruses at a MOI of 0.01. Viral titers were quantitated in SPF embryonated chicken eggs. **(B)** Cytokine expression at the transcription level in primary tree shrew renal cells infected with pdmH1N1, H5N1, or H7N9 influenza viruses. Primary tree shrew cells were either mock-infected or infected with one of the three viruses at a MOI of 1. Total RNAs were prepared at the indicated time points and analyzed by qRT-PCR to quantitate the cytokine mRNA levels. The data shown represent three independent experiments; bars represent the mean ± SD of the three independent experiments (*n* = 3). *p < 0.05.

Next, we evaluated the infectivity of tree shrew primary renal and primary lung cells (TSPRCs or TSPLCs) by infected them with three viruses at a MOI of 0.01 in the presence of trypsin. Supernatants were collected at the indicated time-points for virus titration. As shown in Figure 1A, each virus replicated efficiently in the two cell types. The H5N1 virus titer reached a higher level in both cell types compared with the titers of the pdmH1N1 and H7N9 viruses at 24, 48, and 72 h post-infection (hpi). Virus replication in the TSPLCs reached a peak at 48 hpi, whereas in the TSPRCs, the titers of all three viruses continued to increase during the observation period (72 hpi) and were relatively high compared with those in the TSPLCs. We also assessed the mRNA levels of three major cytokines in the TSPRCs infected with the viruses (Figure 1B). Within the observation period, H7N9 virus induced a sharp increase of IFNβ expression at the mRNA level to about 1,000 folds at 24 hpi, while only 28 folds or even less induced by the H5N1 or pdmH1N1 virus. As for the IFNβ associated genes (IFIT2 and OASL), the H5N1 virus induced an upregulated expression within three viruses at the earliest stage. While at 24 hpi the H5N1 and H7N9 viruses induced higher expression of IFIT2 and OASL, compared with the pdmH1N1 virus. These results indicate that the pdmH1N1, H5N1, and H7N9 influenza viruses can efficiently replicate and activate innate immune responses in primary tree shrew cells.

### PdmH1N1, H5N1, and H7N9 Viruses Efficiently Replicate in Tree Shrews and Cause Subclinical Symptoms

**Figure 2:**
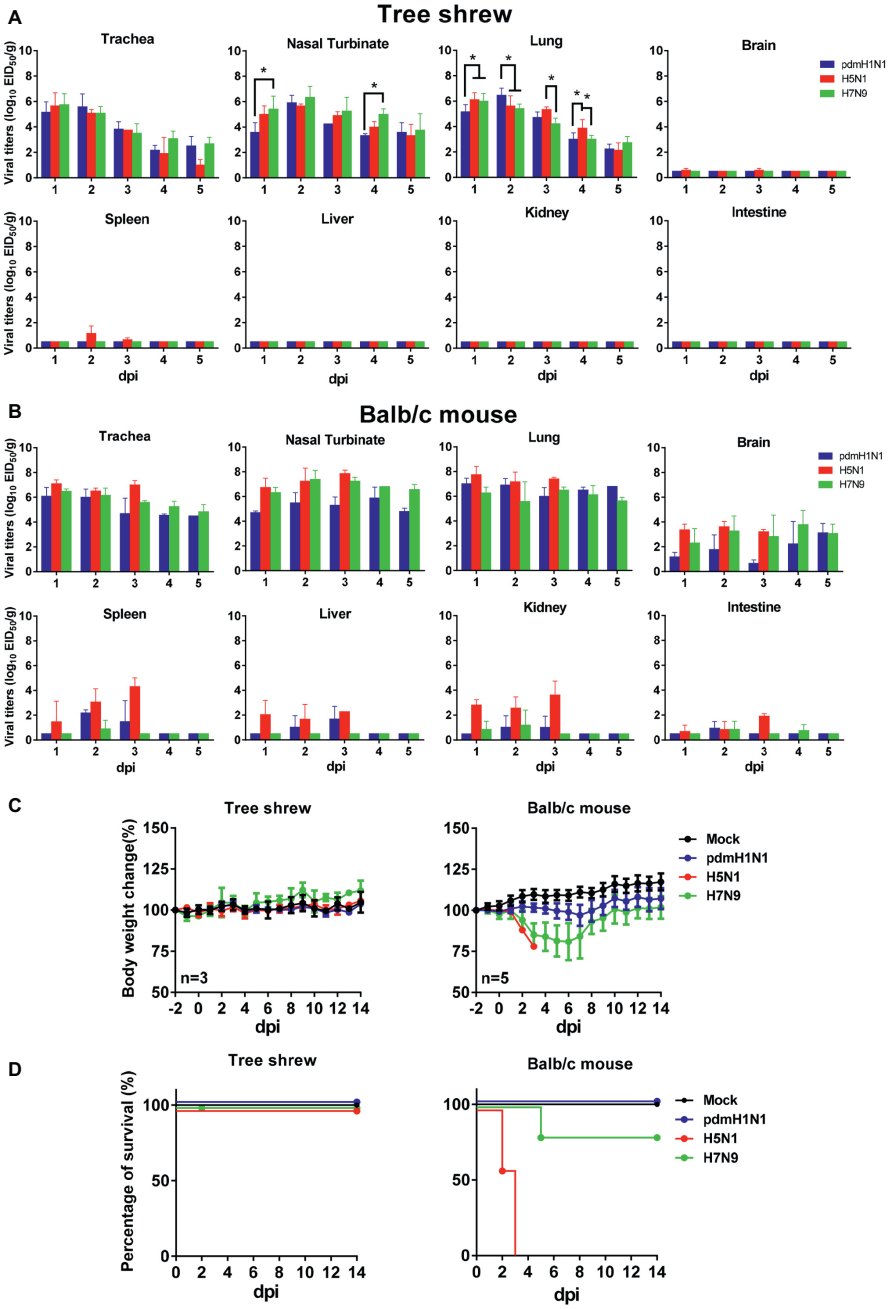
Pathogenicity and virus titers in tissues of animals inoculated with pdmH1N1, H5N1, or H7N9 viruses. Three tree shrews or mice in each group were inoculated with pdmH1N1, H5N1, or H7N9 viruses or were mock-inoculated on day 0. Three tree shrews **(A)** or BALB/c mice **(B)** in each group were euthanized on days 1–5 post-inoculation, and the trachea, nasal turbinate, lung, brain, kidney, spleen, liver, and intestine were collected for viral titration in chicken eggs. Data shown are viral titers (log_10_ EID_50_/g) from three animals; error bars indicate standard deviations. Averages and standard deviations of body weight changes **(C)** and survival **(D)** of three tree shrews in each group were calculated daily. Body weights are presented as percentages of the body weights before virus inoculation. Body weight changes of individual animals on each day after virus inoculation were compared with the average body weight days 2 before virus inoculation. Thereafter, averages and standard deviations of the body weight changes of the animals are indicated in the graphs. The values for body weights are means ± SD from live animals. *p < 0.05.

To evaluate virologic characteristics in tree shrews, we intranasally inoculated tree shrews and balb/c mice (as controls) with pdmH1N1, H5N1, and H7N9 viruses. As shown in Figure 2A, all three influenza viruses replicated well in the respiratory tract. In the tree shrew respiratory tract, the viral titers reached a peak on day 1 or 2 post-inoculation (dpi), with infectious viral loads of over 10^5^ and up to 10^7^ EID_50_/g. The H5N1 virus also replicated in the brains and spleens of the infected tree shrews on 1, 2, and 3 dpi, although the virus titers only ranged from 10^0.58^ to 10^1.5^ EID_50_/g. The viruses were not detected in the spleens or brains of tree shrews inoculated with the pdmH1N1 or H7N9 viruses within the experimental time period. The virus titers declined dramatically from 3 dpi in the trachea and in almost all lung lobes of animals inoculated with the pdmH1N1 and H7N9 viruses, and reached a very low level (<10^3^ EID_50_/g) by 4 dpi. However, the virus titers in these same tissues of animals inoculated with H5N1 virus remained high (>10^5^ EID_50_/g) until 3 dpi, and decreased to a very low level (<10^3^ EID_50_/g) by 5 dpi. As a control, we noted that the three viruses effectively replicated in the respiratory tract of the mice, and even saw systemic replication in the brain, liver, spleen, kidneys, and intestine (Figure 2B). These results indicate that tree shrews are susceptible to H1N1, H5N1, and H7N9 influenza viruses, as are mice, and that the tissue tropism of IAVs in the tree shrew model is mainly restricted in the respiratory tract.

Clinical observation during the course of infection revealed occasional snivel and sneezing. None of the infected tree shrews showed any weight loss during the observation period (Figure 2C). However, the infected mice showed varying degrees of decreased body weight, especially the H5N1-infected mice, which died within 5 days of infection (Figures 2C,D). These data suggest that, compared with the mouse model, tree shrews show a subclinical pathotype on infection with pdmH1N1, H5N1, and H7N9 viruses.

### PdmH1N1, H5N1, and H7N9 Influenza Viruses Induce Pathological Lesions and Inflammatory Responses in the Lung of Tree Shrews

**Figure 3:**
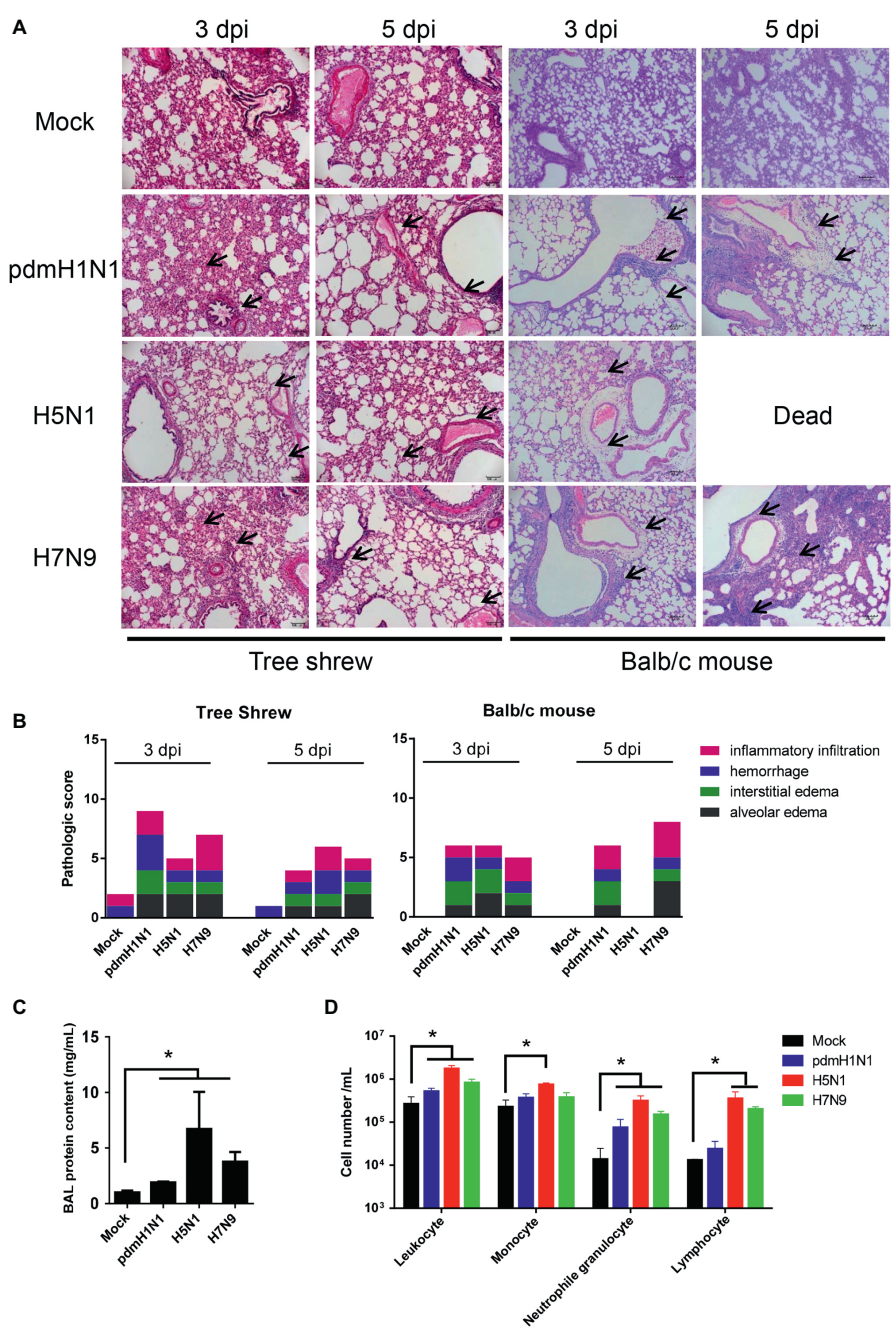
Histopathology and inflammatory response in the lungs of tree shrews inoculated with influenza viruses. **(A)** Representative pathological images of inoculated lungs on days 3 and 5 post-inoculation. Scale bars, 100 μm. **(B)** Histopathological lung lesion scores from tree shrews inoculated with one of the three test viruses. Black arrows indicate edema, hemorrhage, or inflammatory infiltration. **(C)** Protein content was quantified by using a BCA assay with the BALF obtained from tree shrews at day 3 p.i. **(D)** Differential cell counts of BALF obtained from tree shrews at day 3 p.i. The data are presented as means ± SDs for samples of three animals (*n* = 3). *p < 0.05.

We assessed whether histological changes occurred in the lung tissues of tree shrews and mice inoculated with pdmH1N1, H5N1, or H7N9 viruses. The lungs of virus-infected tree shrews at 3 dpi showed obvious alveolar edema, interstitial edema, hemorrhage, and inflammatory infiltration. The inflammation was slightly milder at 5 dpi (Figure 3A). The histopathology score of the infected lungs from tree shrews was as high as 9.0 at 3 dpi and 6.0 at 5 dpi (Figure 3B). Of note, pdmH1N1 induced more severe lesions in infected lungs than H5N1 and H7N9. Lung inflammation in inoculated mice was relatively worse at 5 dpi compared with at 3 dpi, which may have been due to persistent high-level virus loads.

The physiopathology and disease progression induced by influenza virus involve destruction of the pulmonary capillary endothelium and alveolar epithelium as a result of neutrophil, macrophage, and erythrocyte accumulation, as well as protein-rich fluid in the alveolar spaces (Herold et al., 2006). Proteins in the bronchoalveolar lavage fluid (BALF) are usually measured as surrogates for disruption of the alveolar-capillary barrier (Ware and Matthay, 2000). As shown in Figure 3C, the alveolar-capillary barrier integrity following H5N1 virus infection was seriously disturbed, as indicated by the increased BALF protein content. For pdmH1N1 and H7N9 viruses, the BALF protein content was increased to different degrees compared with the mock infection. The total white cell count in the BALF was significantly increased following virus infection, especially for H5N1 virus (Figure 3D). Differential cell counts of BALF from infected tree shrew showed a significant increase in neutrophil and lymphocyte numbers compared with mock-infected tree shrews. These results suggest that influenza viruses induce pathological lesions and inflammatory responses in the lungs of tree shrews.

### PdmH1N1, H5N1, and H7N9 Influenza Viruses Induce Protective Responses Against Homologous Challenge in Tree Shrews

Next, we examined sera from the inoculated tree shrews for antibodies against the influenza viruses by using hemagglutination inhibition (HI) and virus neutralization (VN) tests (Table 3). All tree shrews inoculated with pdmH1N1, H5N1, or H7N9 viruses generated specific antibodies at 3 weeks post-inoculation. The HI antibody against pdmH1N1 virus showed the highest titers in the sera of the inoculated tree shrews, whereas H5N1 virus induced the lowest antibody level in the sera. The VN test showed antibodies from the inoculated tree shrews had high neutralizing activity against the corresponding viruses. Not surprisingly, pre-inoculation sera were negative for influenza virus-specific antibodies by the HI and VN tests.

**Table 3:** Seroconversion of tree shrews pre- and post-inoculated with different influenza viruses.

Tree shrews inoculated with	Seroconversion (positive/total)^a^			
	Pre-inoculation sera		Post-inoculation sera	
	HI titer	VN titer	HI titer	VN titer
pdmH1N1	–^b^	–	3/3 (80–160)	3/3 (447–1,778)
H5N1	–	–	3/3 (20–80)	3/3 (158–447)
H7N9	–	–	3/3 (80–160)	3/3 (56–447)

a *Sera were collected from the animals at 2 days pre- or 3 weeks after the virus inoculation. The range of antibody titers obtained is indicated in parentheses*.

b *No antibody titer was detected*.

**Figure 4:**
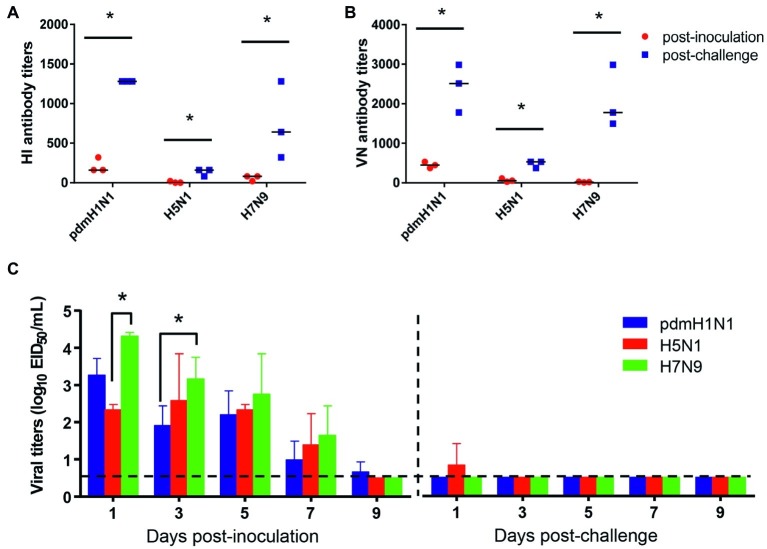
Antibody response and virus shedding in tree shrews inoculated or challenged with influenza viruses. **(A,B)** Serum antibody titers were measured by using the HI method and virus neutralization test with serum collected at 14 days post-inoculation (red dots) and 14 days post-challenge (blue dots). **(C)** Nasal washes for virus shedding detection were collected every other day from tree shrews from day 2 post-inoculation or -challenge. Viral titers in nasal wash samples from each of three individual tree shrews are expressed as log_10_ EID_50_/ml. The data are presented as means ± SDs for nasal wash samples of three animals (*n* = 3). *p < 0.05. Horizontal dashed lines indicate the lower limit of virus detection.

To test the protection efficacy against homologous challenge, we first inoculated tree shrews with 10^6^ EID_50_/ml of pdmH1N1, H5N1, or H7N9 virus and then challenged them with 10^6^ EID_50_/ml of homologous viruses at 3 weeks after inoculation. During the observation period, no clinic symptoms appeared in the tree shrews. Sera were collected at 14 days post-inoculation and 14 days post-challenge for HI and VN tests, which revealed a significant increase in the antibody level in each challenge group (Figures 4A,B). In addition, considerable virus shedding was detected in the nasal wash collected from the inoculated tree shrews from day 1 to day 7 post-inoculation, whereas almost no virus was detected in any of the nasal wash collected from the challenged tree shrews within the observation period (Figure 4C). These data suggest that single dose inoculation of tree shrews induced protective immunity against homologous influenza virus challenge.

### PdmH1N1 and H7N9, but not H5N1, Influenza Viruses Are Transmissible in Tree Shrews

**Figure 5:**
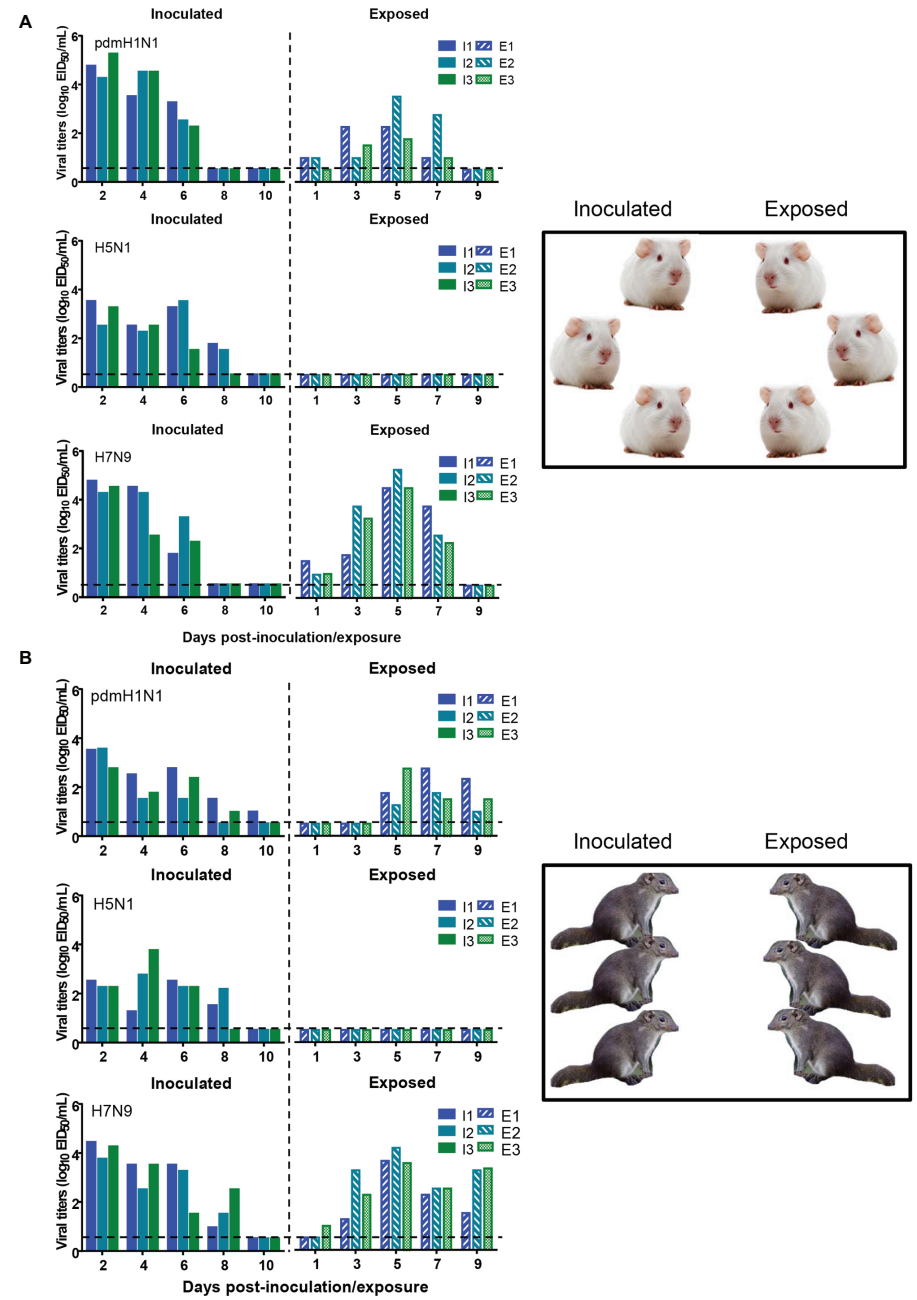
Transmission of pdmH1N1, H5N1, and H7N9 viruses in guinea pigs **(A)** and tree shrews **(B)**. Groups of three guinea pigs or tree shrews were inoculated with 10^6^ EID_50_ of the indicated viruses. The next day, the inoculated animals were cohoused with three guinea pigs or tree shrews. Nasal washes for virus shedding detection were collected every other day from all animals from day 2 of the initial infection. Viral titers in nasal wash samples from each of three individual inoculated animals (solid bars, left) and contact animals (dotted/diagonal bars, right) are expressed as log_10_ EID_50_/ml. Each color bar represents a value from an individual animal. Horizontal dashed lines indicate the lower limit of virus detection. I or E means Inoculated or Exposed, respectively.

To test whether the three different influenza viruses can transmit in the tree shrew model, we evaluated their transmissibility among tree shrews by using a direct-contact approach, and employed a well-defined transmission model (guinea pig) for influenza virus as a control. The pre-inoculation sera from each animal at day 2 before inoculation were negative for influenza virus-specific antibodies by the HI and VN tests (data not shown). In the transmission experiment for pdmH1N1 virus, virus efficiently replicated in the upper respiratory tract of guinea pigs with titers of ≥10^4.25^, ≥ 10^3.5^, and ≥ 10^2.25^ EID_50_/ml on days 2, 4, and 6 post-inoculation, respectively. Low titers of virus were detected in the nasal washings of exposed guinea pigs from day 1 p.e. (Figure 5A). Only one of three exposed guinea pigs seroconverted, with an HI titer of 80 and a VN titer of 531, whereas all pdmH1N1 virus-inoculated guinea pigs possessed HI titers ≥80 and VN titers ≥376 (Table 4). A similar trend was observed in the tree shrew groups; however, viruses were detectable in three nasal wash samples from day 5 p.e. in the exposed tree shrews (Figure 5B). Serum antibodies from two of three exposed tree shrews were seroconverted with HI titers of 40 and 320 and VN titers of 266 and 1,259 against pdmH1N1, respectively, whereas all inoculated tree shrews possessed HI titers ≥80 and VN titers ≥447 (Table 4). In the H7N9 transmission experiment, not surprisingly, H7N9 virus was readily transmitted from the three inoculated guinea pigs to the three contact ones with viral loads of up to 10^4.5^ EID_50_/mL on day 5 p.e. (Figure 5A). Sera from all guinea pigs exposed to H7N9 virus were seroconverted with HI titers of 40–160 and VN titers of 79–447, whereas the inoculated guinea pigs possessed higher antibody titers with HI titers of 80–160 and VN titers 224–531 (Table 4). H7N9 virus also transmitted from the inoculated tree shrews to the contact animals (Figure 5B). Detectable virus persisted in nasal wash samples until day 9 p.e., while the viral load reached its peak on day 5 at 10^4.17^ EID_50_/ml (Figure 5B). Sera from the exposed tree shrews were all seroconverted with HI titers of 20–80 and VN titers of 94–112, whereas the inoculated tree shrews induced higher antibody levels with HI titers of 80–160 and VN titers of 56–447 (Table 4). However, avian-origin H5N1 did not transmit in guinea pigs or tree shrews by direct contact in the present study (Figures 5A,B). No virus was detected in any of the nasal wash samples collected from the exposed guinea pigs or tree shrews within the observation period, and sera collected from the contact animals were negative against H5N1 virus at 21 days post-exposure (Figures 5A,B and Table 4). Together, these results indicate that pdmH1N1 and H7N9 viruses can transmit among guinea pigs and tree shrews in a direct contact model and that H7N9 has stronger transmissibility among guinea pigs than tree shrews compared with pdmH1N1 and H5N1 viruses.

**Table 4:** Seroconversion of guinea pigs and tree shrews inoculated with or exposed to different influenza viruses.

Virus	Animals	Seroconversion (positive/total)^a^			
		Inoculated		Exposed	
		HI titer	VN titer	HI titer	VN titer
H1N1	Guinea pig	3/3 (80–320)	3/3 (376–631)	1/3 (80)	1/3 (531)
H5N1	Guinea pig	3/3 (20–40)	3/3 (112–447)	0/3	0/3
H7N9	Guinea pig	3/3 (80–160)	3/3 (224–531)	3/3 (40–160)	3/3 (79–447)
H1N1	Tree shrew	3/3 (80–160)	3/3 (447–1,778)	2/3 (40–320)	2/3 (266–1,259)
H5N1	Tree shrew	3/3 (20–80)	3/3 (158–447)	0/3	0/3
H7N9	Tree shrew	3/3 (80–160)	3/3 (56–447)	3/3 (20–80)	3/3 (94–112)

a *Sera were collected from the animals at 3 weeks after the virus inoculation or exposure; these animals are the same ones that were used for the transmission studies shown in Figure 5. The range of antibody titers obtained was indicated in parentheses*.

### Interspecies Transmission of H7N9 Influenza Virus From Tree Shrews to Guinea Pigs

**Figure 6:**
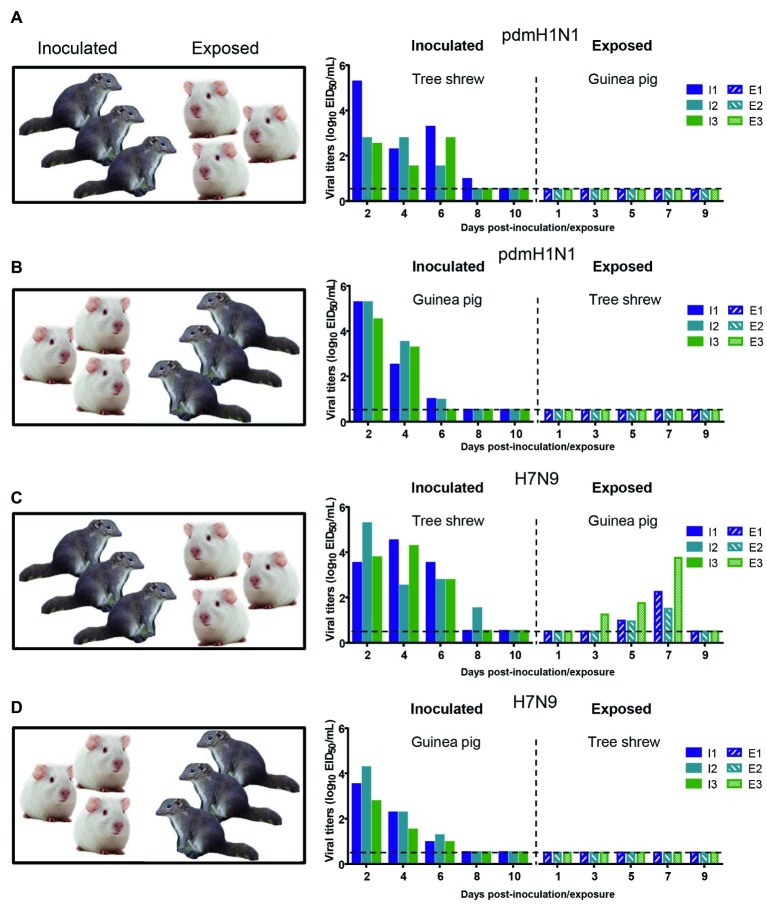
Interspecies transmission of pdmH1N1 and H7N9 viruses between guinea pigs and tree shrews. Three animals were inoculated with 10^6^ EID_50_ of pdmH1N1 virus **(A,B)** or H7N9 virus **(C,D)**, respectively. The next day, for **A** and **C**, the inoculated tree shrews were cohoused with three naive guinea pigs; for **B** and **D**, the inoculated guinea pigs were cohoused with three naive tree shrews. Nasal washes for virus shedding detection were collected every other day from all animals from day 2 of the initial infection. Viral titers in nasal wash samples from each of three individual inoculated animals (solid bars, left) and contact animals (dotted/diagonal bars, right) are expressed as log_10_ EID_50_/ml. Each color bar represents a value from an individual animal. Horizontal dashed lines indicate the lower limit of virus detection. I or E means Inoculated or Exposed, respectively.

The interspecies transmission of IAVs poses a potential threat to humans and animals (Shi et al., 2018; WHO, 2018; Zeng et al., 2018). Based on the transmissibility of pdmH1N1 and H7N9 viruses in guinea pigs and tree shrews described above, we next sought to test whether these two viruses could transmit from guinea pig to tree shrew, or vice versa. For pandemic H1N1 virus, as shown in Figures 6A,B, the virus was not detected in the respiratory tract of the exposed guinea pigs or tree shrews, although pdmH1N1 was detectable in the nasal wash from the inoculated guinea pigs and tree shrews, suggesting that pdmH1N1 is not transmissible from tree shrews to guinea pigs, or vice versa. For H7N9 virus, viral shedding was detected on days 3–7 p.e. from the three exposed guinea pigs housed with the tree shrews inoculated with H7N9 virus (Figure 6C), but the corresponding interspecies transmission model was not established (Figure 6D). Consistent with the above, in the four groups of exposed animals, only the serum antibodies from two of the exposed guinea pigs co-housed with the H7N9-inoculated tree shrews were seroconverted with HI titers of 80 and VN titers of 316 (Table 5). These results suggest that the H7N9 virus, but not pdmH1N1, is transmissible from tree shrews to guinea pigs.

**Table 5:** Seroconversion of animals in the interspecies transmission study.

Virus	Seroconversion (positive/total)^a^					
	Inoculated			Exposed		
	Animals	HI titer	VN titer	Animals	HI titer	VN titer
pdmH1N1	Tree shrew	3/3 (80–640)	3/3 (188–376)	Guinea pig	0/3	0/3
pdmH1N1	Guinea pig	3/3 (40–320)	3/3 (40–266)	Tree shrew	0/3	0/3
H7N9	Tree shrew	3/3 (20–40)	3/3 (79–188)	Guinea pig	2/3 (80)	2/3 (316)
H7N9	Guinea pig	3/3 (80–160)	3/3 (224)	Tree shrew	0/3	0/3

a *Sera were collected from the animals at 3 weeks after virus inoculation or exposure; these animals are the same ones that were used for the transmission studies shown in Figure 6. The range of antibody titers obtained was indicated in parentheses*.

## Discussion

Previous studies have demonstrated that H1N1 and H9N2 influenza viruses replicate in the upper respiratory tract of tree shrews, which showed moderate respiratory symptoms and pathological signs (Yang et al., 2013; Li et al., 2018). Furthermore, while our manuscript was in preparation, Sanada and colleagues documented the pathogenicity of H5N1 and H7N9 IAVs in tree shrews (Sanada et al., 2019). In the present study, we performed a more comprehensive and in-depth investigations and comparison of the suitability of the tree shrew as an animal model for the study of three different IAV subtypes from various species. Three influenza viruses, pdmH1N1, H5N1, and H7N9, were isolated from humans and birds, respectively, and represent a severe threat to human health. Our integrated evaluation of the tree shrew model enriched the study about the infectivity and transmissibility of animal influenza viruses in the field.

Numerous human and non-human cell lines have been used to study the pathogenicity, replication, and innate immune response of influenza viruses (Steel et al., 2009; Elbahesh et al., 2014; Wang et al., 2018). Compared with cell lines, primary cells could reflect viral infectivity and pathogenicity *in vivo* from another facet since primary cells from animal models possess their inherent biological properties. For instance, primary tree shrew cells have been used as an *in vitro* platform for HBV, HCV, and ZIKV (Glebe et al., 2003; Feng et al., 2017; Zhang et al., 2019). In the present study, we demonstrated that primary renal and lung epithelial cells from the tree shrew were suitable to study the infectivity and innate immune response induced by pdmH1N1, H5N1, and H7N9 viruses (Figure 1).

The clinical manifestations of influenza virus are various in humans. Most infected people develop a fever, cough, runny nose, loss of appetite, etc. In the worst cases, influenza virus may cause pneumonia, respiratory distress syndrome, and even death (Carrat et al., 2008). Previous studies have demonstrated that tree shrews infected with H1N1, H7N9, or H9N2 virus showed moderate respiratory symptoms and pathological signs (Yang et al., 2013; Li et al., 2018; Sanada et al., 2019). Sanada et al. reported that H5N1 infection induced severe clinical symptoms including pneumonia, fever, weight loss, and death in tree shrews (Sanada et al., 2019). However, in our study, tree shrews infected with H1N1, H5N1, or H7N9 virus did not show significant weight loss or obvious clinical signs (Figure 2). The reasonable explanation for this discrepancy is the origin of the virus strains. The H5N1 virus used in our study was isolated from chickens, whereas the H5N1 virus (A/Vietnam/UT3040/2004) used in Sanada’s study was from a human patient and possessed adaptive mutations (PB2 E627K, G309D) that can lead to enhanced virulence in mammals. In addition, pdmH1N1, H5N1, and H7N9 viruses possessed different abilities to replicate in the non-respiratory organs of mice and tree shrews because of a distinct tissue tropism. (Figure 2), suggesting that these three IAVs could be used for the study on the respiratory tract-related diseases in the tree shrew model. Finally, in the tree shrew, we observed obvious pulmonary lesions and inflammatory responses and a large number of neutrophils, monocytes, and proteins in the BALF, which similarly appeared in many human clinical cases infected with influenza viruses (Yokoyama et al., 2010; Shen et al., 2015). Therefore, tree shrews have potential as a mammalian model for the study of influenza virus pathogenesis.

Vaccination is the most effective and cost-effective healthcare intervention to prevent influenza infection. However, the frequent antigenic shift and drift of influenza virus results in mismatches between the vaccine and circulating influenza virus strains, making it important to assess vaccine potency promptly in a suitable animal model (Singanayagam et al., 2018). Animal models not only play an important role in our understanding of viral pathogenicity, but also serve as a platform for the evaluation of vaccine candidates and new therapeutics (Margine and Krammer, 2014). In the present study, tree shrews induced an ideal humoral immune response upon infection with different influenza viruses. Infected tree shrews were protected against a second challenge with the homologous virus. Although the magnitude of the antibody responses in the tree shrews was variable, this variation appears to be due to the different viruses, which would assist us in selecting appropriate vaccine candidate strains. Therefore, we believe that the tree shrew model would be helpful for evaluating the efficacy of vaccines for the prevention of influenza infection.

Studies of influenza virus transmission in mammals are essential to assess potential public health risks and for pandemic preparedness. Previous work has shown that both strong binding to human like α2,6 sialic acid receptors and potent replication in the respiratory tract are necessary for efficient transmission in mammals (Schrauwen and Fouchier, 2014). Previous findings indicate that human-like α2,6 sialic acid receptors are mainly distributed in the nasal mucosa, trachea, and bronchus of tree shrews, and avian-like ɑ2,3 sialic acid receptors are primarily distributed in the trachea, bronchiole, and alveolus of tree shrews (Yang et al., 2013). In our study, the H5N1 avian influenza virus prior to adaptation in mammals had no affinity for α2,6 sialic acid receptors (Table 2; Supplementary Figure S1), it is not surprising that the H5N1 virus failed to transmit in either the guinea pig or tree shrew model, consistent with previous studies in other animal models (mouse, guinea pig, and ferret) (Lowen et al., 2006; Maines et al., 2006). In contrast, the low pathogenic pdmH1N1 and H7N9 viruses tested in this study transmitted efficiently between tree shrews with high-titer virus shedding in nasal wash and seroconversion of directly inoculated and exposed groups. To our knowledge this represents the first reporting of an unadapted H7N9 influenza virus being transmitted among tree shrews by direct contact. Further studies should examine whether aerosol spread of H7N9 influenza virus occurs in tree shrews.

The risk of a new influenza pandemic increases with repeated interspecies transmission events (Wang et al., 2013; Zhang et al., 2017). In early 2013, a novel reassortant a*via*n-origin influenza A (H7N9) virus crossed the species barrier and caused the first human infection (Gao et al., 2013; Zhang et al., 2013a). So far, five waves of human H7N9 infection have caused 1,567 cases, with a fatality rate of approximately 39% (WHO, 2018; Zeng et al., 2018). Moreover, in 2017, H7N9 highly pathogenic influenza viruses were detected in poultry and humans (Shi et al., 2017, 2018). H7N9 viruses easily acquire the PB2 E627K or D701N mutation upon replication in mammals (Chen et al., 2013; Shi et al., 2017). Additionally, the viral PA protein and host ANP32A protein are crucial for the emergence of PB2 E627K during adaptation of H7N9 avian influenza viruses to humans (Liang et al., 2019). Nevertheless, there is currently no evidence that the H7N9 influenza virus can spread from person to person. Among the mammalian hosts, pigs, possessing both avian-like and human-like receptors in the respiratory tract, are susceptible to infection with various influenza viruses and have been deemed to be “mixing vessels” (Hass et al., 2011). Two studies reported that H7N9 isolate was able to infect and easily adapted to pigs, and the six internal gene combination of H7N9 virus, which are probably derived from H9N2 viruses, functions in viral replication and transmission in mammals (Zhu et al., 2013; Li et al., 2014; Xu et al., 2014). Therefore, human or avian H7N9 influenza virus could be potentially introduced into swine population and initiate a multiple gene reassortment in nature. In the present study, the tree shrew-guinea pig interspecies transmission model can mimic the transmission between porcine and human to some extent. Our data suggest that H7N9 virus can cross the species-barrier from tree shrews to guinea pigs (Figure 6), which implies the importance of implementing biosecurity measures in pig farms and the necessity of changing the co-habitation pattern of different livestock and poultry to prevent interspecies transmission. Future studies will examine why this interspecies transmission is unidirectional and why H7N9 virus rather than pdmH1N1 virus was able to transmit interspecies. The tree shrew is thus a promising tool for studies on the interspecies transmission of H7N9 influenza virus.

In summary, our comprehensive study suggests that tree shrew is susceptible to different subtypes of IAVs that replicate efficiently in the both the upper and lower respiratory tracts prior to adaptation. Virus inoculation may protect tree shrews against challenge from homologous virus. Importantly, this study revealed that unadapted H7N9 virus is transmissible among tree shrews and interspecies transmission can occur from tree shrews to guinea pigs *via* the direct-contact mode. Therefore, taken together with all of the merits of tree shrews in our or previous studies, we believe that tree shrews could be a promising alternative animal model for the study of influenza virus pathogenesis and transmission, and for assessments of antiviral agents and vaccines.

## Data Availability Statement

All datasets generated for this study are included in the article/Supplementary Material.

## Ethics Statement

The animal study was reviewed and approved by Animal Ethics Committee of Lanzhou Veterinary Research Institute, Chinese Academy of Agricultural Sciences.

## Author Contributions

SX and QZ conceived this study. SX, XL, JY, ZW, YJ, LH, and LW conducted the experiments. SX and QZ analyzed the data and wrote the paper. SX and XL contributed equally to this work.

### Conflict of Interest

The authors declare that the research was conducted in the absence of any commercial or financial relationships that could be construed as a potential conflict of interest.
