# Supplementary figures and images for: Comparative Pathogenicity and Transmissibility of Pandemic H1N1, Avian H5N1, and Human H7N9 Influenza Viruses in Tree Shrews
Source: Front Microbiol. 2019 Dec 20;10:2955. doi: 10.3389/fmicb.2019.02955 (PMC6933948; doi:10.3389/fmicb.2019.02955)

## Slide 1
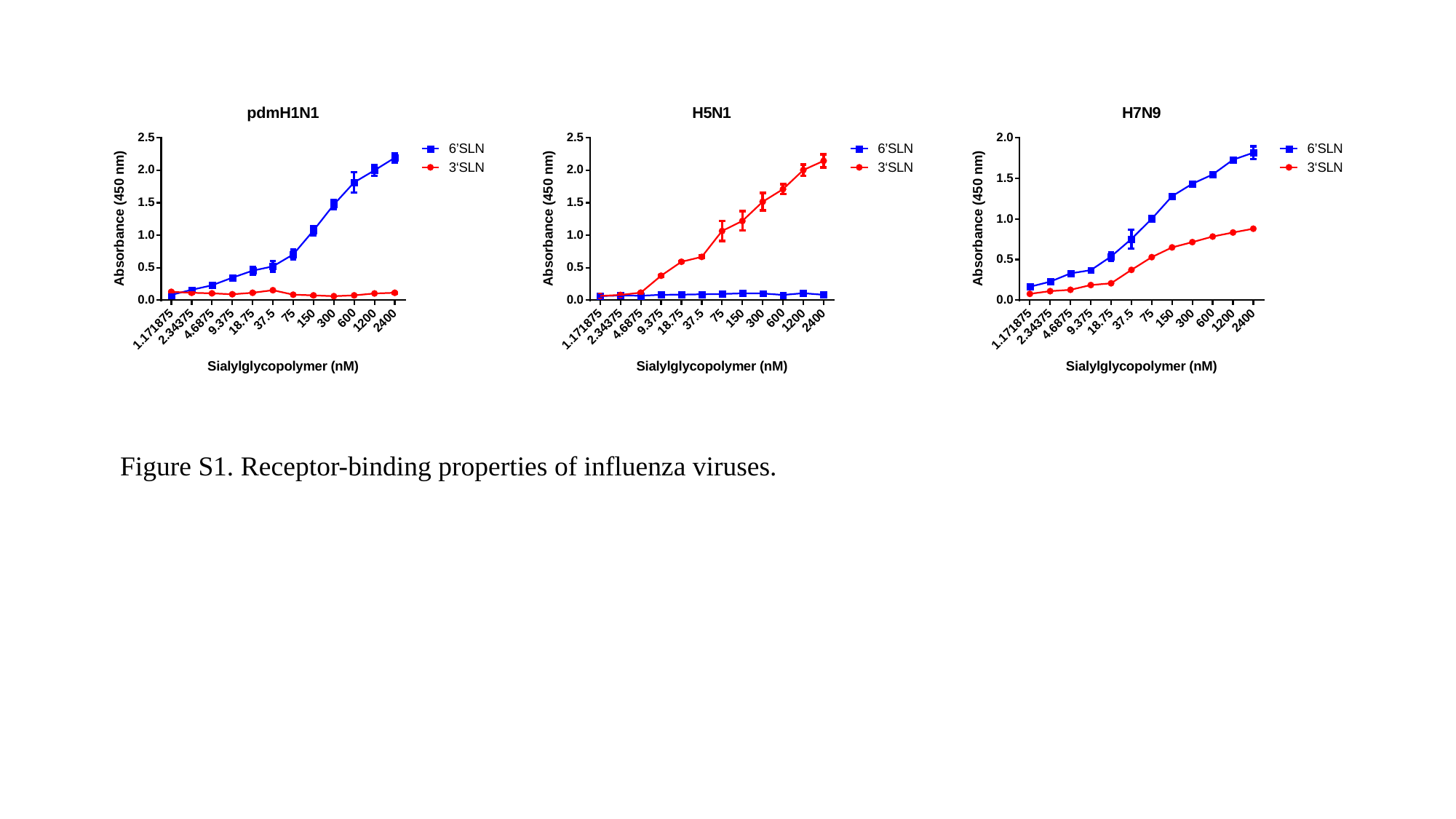

Figure S1. Receptor-binding properties of influenza viruses.

Supplement: Supplementary file 1 [file Presentation_1.PPTX]
